# Estimates of hepatitis B virus prevalence among general population and key risk groups in EU/EEA/UK countries: a systematic review

**DOI:** 10.2807/1560-7917.ES.2023.28.30.2200738

**Published:** 2023-07-27

**Authors:** Sandra Bivegete, Anna L McNaughton, Adam Trickey, Zak Thornton, Becky Scanlan, Aaron G Lim, Lina Nerlander, Hannah Fraser, Josephine G Walker, Matthew Hickman, Peter Vickerman, Helen Johnson, Erika Duffell, Ellen Brooks-Pollock, Hannah Christensen

**Affiliations:** 1 Population Health Sciences, Bristol Medical School, University of Bristol, United Kingdom; 2 Health Protection Research Unit (HPRU) in Behavioural Science and Evaluation, Bristol, United Kingdom; 3 European Centre for Disease Prevention and Control (ECDC), Stockholm, Sweden

**Keywords:** Epidemiology, hepatitis B Virus, prevalence, risk groups, systematic review

## Abstract

**Background:**

The burden of chronic hepatitis B virus (HBV) varies across the European Union (EU) and European Economic Area (EEA).

**Aim:**

We aimed to update the 2017 HBV prevalence estimates in EU/EEA countries and the United Kingdom for 2018 to 2021.

**Methods:**

We undertook a systematic review, adding to HBV prevalence estimates from an existing (2005–2017) database. Databases were searched for original English-language research articles including HBV surface antigen prevalence estimates among the general population, pregnant women, first-time blood donors (FTB), men who have sex with men (MSM), migrants and people in prison. Country experts contributed grey literature data. Risk of bias was assessed using a quality assessment framework.

**Findings:**

The update provided 147 new prevalence estimates across the region (updated total n = 579). Median HBV prevalence in the general population was 0.5% and the highest was 3.8% (Greece). Among FTB, the highest prevalence was 0.8% (Lithuania). Estimates among pregnant women were highest in Romania and Italy (5.1%). Among migrants, the highest estimate was 31.7% (Spain). Relative to 2017 estimates, median prevalence among pregnant women decreased by 0.5% (to 0.3%) and increased by 0.9% (to 5.8%) among migrants. Among MSM, the highest estimate was 3.4% (Croatia). Prevalence among people in prison was highest in Greece (8.3%) and the median prevalence increased by 0.6% (to 2.1%).

**Conclusions:**

The HBV prevalence is low in the general population and confined to risk populations in most European countries with some exceptions. Screening and treatment should be targeted to people in prison and migrants.

**Key public health message**

**What did you want to address in this study?**

The World Health Organization aims to diagnose 90% and treat 80% of all people with chronic hepatitis B virus (HBV) infection by 2030. This study aimed to provide up-to-date estimates of the proportion of the population who are chronically infected with HBV in the European Union, European Economic Area and the United Kingdom. We looked at the general population, pregnant women, first time blood donors and several high-risk groups.

**What have we learnt from this study?**

Compared with previous estimates, infection prevalence declined in the general population, men who have sex with men (MSM) and pregnant women, maybe as a result of increased vaccination and improved screening and treatment. The proportion infected with HBV among prison populations and migrants was particularly high in several studies. There was not much information for eastern Europe, particularly among MSM and migrant groups.

**What are the implications of your findings for public health?**

Understanding which populations are affected by HBV, and the level of infection in these groups is crucial for testing and prevention strategies. Public health interventions should target migrants and people in prison as a priority. The updated estimates can help inform policymakers to advocate for the prevention, diagnosis, and treatment in those most vulnerable, supporting efforts to reach the elimination goals.

## Introduction

Infection with hepatitis B virus (HBV) can cause acute and chronic hepatitis B infection (CHB). Infections with HBV are frequently asymptomatic and often only detected when people are screened or tested for other reasons. The World Health Organization (WHO) estimated in 2019 that 296 million people worldwide lived with CHB and that only 10.5% of these individuals were aware of their infection [1,2]. Progression of CHB can lead to severe liver disease with outcomes such as cirrhosis and liver cancer occurring in 20–30% of chronically infected individuals [3].

The virus spreads through blood, semen and other bodily fluids [4]. In regions where HBV is highly endemic (> 8% prevalence [5]), transmission of the virus often occurs vertically from mother to child as well as horizontally through close contacts during infancy [4]. In the European Union and European Economic Area (EU/EEA) and the United Kingdom (UK), transmission via sexual contact and nosocomial routes are commonly reported and thought to be responsible for more than half of all acute HBV cases in the area [6]. The European Region is considered low-prevalence for HBV (< 2% [5]), with overall prevalence estimated to be 0.9% in 2016 [7], although there are marked geographical differences, with prevalence generally higher in eastern and southern Europe, and lower in western, central and northern Europe [8]. Hepatitis B remains a major public health problem in the EU/EEA/UK, with an estimated 4.7 million individuals living with CHB [7], causing ca 43,000 deaths annually in 2019 [1]. However, HBV is not widely endemic in the EU/EEA/UK and most frequently affects people who have migrated with CHB from high endemicity areas and people from specific risk groups [6].

The HBV prevalence estimates in the EU/EEA/UK region are typically derived from the general population and first-time blood donor (FTB) data on the prevalence of hepatitis B surface antigen (HBsAg), a widely used biomarker for chronic/active HBV infection. Other population groups contributing to the national HBV prevalence estimates in EU/EEA/UK countries are known to have high prevalence, such as migrant populations from countries with a high burden of CHB, men who have sex with men (MSM), people who inject drugs (PWID) and people in prison [8]. These risk groups may be contributing substantially to the overall HBV prevalence in the EU/EEA/UK and should be considered when estimating the general population prevalence.

The United Nations Members States adopted the Sustainable Development Goals in 2015, and one of the health targets is to combat viral hepatitis as a public health issue [9]. To achieve this, the WHO Global Health Sector Strategy (GHSS) has set ambitious elimination targets aiming to reduce CHB incidence by 90% and mortality due to viral hepatitis by 65% by 2030 compared with 2015 [10]. Therefore, the European Centre for Disease Prevention and Control (ECDC) has developed a monitoring programme to support countries in the EU/EEA in assessing progress towards the hepatitis elimination targets, and to help identify gaps in responses. The ECDC conducted systematic reviews of peer-reviewed EU/EEA/UK serosurvey studies, published in 2016 [7], and in 2017 conducted an update of the HBV database [11] to obtain an assessment of the prevalence of HBV in the region among the general population, pregnant women, and key risk groups [12,13]. The existing database contains peer-reviewed publications published between 2005 and 2017. Here, we aim to update HBV prevalence estimates in EU/EEA/UK countries with data derived from published and grey literature between 2018 and 2021.

## Methods

### Search strategy

We undertook a systematic review of HBV prevalence estimates for the EU/EEA/UK in the general population and proxy groups for the general population (FTB and pregnant women) and key risk population groups (MSM, migrant populations and people in prison) and reported the findings using PRISMA guidelines [14]. Definitions of each subgroup can be found in Box 1. We searched PubMed, Embase, and Cochrane Library bibliographic databases for original research articles in English, published between 1 January 2018 and 24 February 2021. We did not perform any searches of studies published in languages other than English.

Box 1 **List and definition of population groups included in the systematic review on hepatitis B virus prevalence estimates, EU/EEA/UK**

- **General Population:** people living in a defined geographical area (all ages or adults only), excluding specific low-risk populations such as children; patients attending community and primary care settings, excluding hospitalised patients; workforce or specific professional groups (i.e. workplace screening) excluding healthcare workers and specific recreational/sports-related population subgroups;
- **Prisoners:** prison inmates and people incarcerated in custodial or prison settings including youth detention centres, excluding formerly incarcerated populations and people in other non-custodial closed/fixed institutions (such as secure psychiatric hospitals); psychiatric prison hospital inmates (i.e. people with severe mental illness that are serving custodial sentences) are included;
- **MSM:** men who have sex with men;
- **Pregnant women:** pregnant women undergoing antenatal care screening;
- **Blood donors:** first-time blood donors (pre-screened and non-pre-screened);
- **Migrants:** foreign-born migrants, not including Roma and other minorities, refugees, asylum seekers and their children.

EEA: European Economic Area; EU: European Union; UK: United Kingdom.This box was reproduced from the 2016 ECDC systematic review [7].

The search strategy was adapted from the existing systematic review [7] by applying a combination of Medical Subject Headings (MeSH), Emtree syntax and free-text searching. Natural vocabulary on HBV prevalence, population subgroups and geographic terms were implemented in the search. Further details of the search strategy are provided in Supplementary Tables S1–S3.

### Selection process

Only studies reporting data from EU/EEA/UK countries were selected. Consistent with the previous review [7], 31 countries were included in the analysis, consisting of 27 EU countries, three EEA countries (Norway, Iceland and Liechtenstein) and the UK. The full list of inclusion and exclusion criteria are provided in Box 1 and 2.

Box 2 **Inclusion and exclusion criteria for studies in the systematic review on hepatitis B virus prevalence estimates, EU/EEA/UK**

**Inclusion category**

- Only studies reporting original data; any relevant review articles identified during the search had their reference lists checked for additional articles not captured by the literature search;
- Articles published between 1 January 2018 and 24 February 2021, reporting data from populations sampled in 2000 or later, including studies with data collection ending after 2000 (irrespective of start date);
- Articles reporting HBsAg prevalence in humans;
- Articles reporting data from the general population and/or key risk groups (Box 1).

**Exclusion category**

- Articles falling outside the specified sampling period or publication date range;
- Articles reporting data on countries outside the EU/EEA/UK only; articles reporting data on the general population or pregnant women in countries with a population of > 5 million inhabitants, but with a sample size < 100 participants or with a sample size < 50 participants for countries with a population of < 5 million;
- Articles not reporting data on HBsAg prevalence and if virological markers tested for were not specified;
- Articles not reporting data on HBsAg prevalence and if virological markers tested for were not specified;
- Articles reporting data on specific key risk groups only: acute/chronic liver disease patients, in-/outpatients, people with haemophilia, healthcare workers, military recruits, children;
- Articles reporting modelled data only^a^; articles reporting only data from a study not conducted in humans, environmental studies, technology assessments (studies on diagnostic and/or laboratory methods); opinion papers, editorials, guidelines or recommendations, perspectives, and correspondence articles, systematic reviews, or meta-analyses.

EEA: European Economic Area; EU: European Union; HBsAg: hepatitis B virus surface antigen; MSM: men who have sex with men; UK: United Kingdom.
^a^ Grey literature reporting modelled estimates for countries were included only if no other data was available. The Box was adapted from the 2016 ECDC systematic review [7].

### Data collection process

Publications were catalogued and de-duplicated using Endnote X9. Titles and abstracts were screened to identify articles containing relevant information. Full-text of articles not written in English were translated using Google Translate. 

Studies were included if they met the inclusion criteria (Box 2) and reported a HBsAg prevalence. To assess concordance before undertaking the full-text screening, a random 10% of articles were selected for double screening of titles and abstracts. One reviewer (SB) screened 10% (n = 413) of the articles, with another two reviewers (BS and ZT) assisting with double screening, and concordance was verified. SB full-text-screened and extracted the data with double extraction of the data undertaken by BS and ZT. If discrepancies occurred, verbal discussion of results was arranged between reviewers. In case of disagreements, third reviewers (JGW and ALM) were consulted to resolve and finalise results.

Studies published in more than one article were extracted only once, and the earliest dated publication was used. When the earliest publication was a conference abstract which was later published as a complete study, we included the full-text article. External grey literature was obtained through contact with the ECDC National Focal Points for Hepatitis, who provided any relevant published and unpublished data they were aware of that had not been captured through the literature search.  Relevant data from each study were extracted for a predefined set of variables covering study characteristics, study population details, prevalence of HBsAg and entered directly into an Excel spreadsheet. The complete list of variables is provided in the earlier 2016 ECDC report [7].

### Risk of bias

To assess the risk of bias in the studies, we adapted a previously developed quality assessment framework [7]. The framework identified key sources of bias hereafter referred to as ‘domains’ in the different risk groups. A scoring system was developed to distinguish between highest (lowest score) and lowest (highest score) risk of bias. For the general population, four domains (age, sex, sampling method, population coverage) were identified, and given a score between 0 and 6. For studies on pregnant women, two domains (sampling method, population coverage) were identified resulting in a score between 0 and 3. Studies on MSM resulted in a score between 0 and 2 in one domain (sampling venue coverage). For people in prison, five domains (age, sex, proportion PWID, sampling method, population coverage) were identified, generating a risk score between 0 and 6. Scores were totalled and considered criteria for high/low risk of bias.

Since previous ECDC reviews of HBV prevalence had not assessed the risk of bias among migrant populations, we used a different approach to assess the quality of these extracted studies, adapted from another systematic review of HBV prevalence [15] (see Supplementary Tables S4–S8 for more details on the scoring of studies in each population group).

### Data synthesis

We combined the newly identified data from our review with data from the existing database [11] to provide CHB prevalence estimates by risk group, proxy population and the general population. Due to the heterogeneity across individual studies, we used a descriptive synthesis approach to combine results within countries and risk groups. To understand prevalence trends in the new estimates versus existing estimates, each group was tabulated as medians and interquartile ranges.

### Weighted prevalence estimates

The data from the existing [11] and updated study resulted in multiple studies per country in each population group. Prevalence estimates were weighted by study population size to determine the weighted country prevalence and attenuated for the high prevalence reported from smaller population size estimates in each country. Weighted average estimates from the existing [11] and updated database with low risk of bias for each country per population group were pooled together. Population size weights per country were used to determine the weighted country prevalence in the population groups which were used to generate heat maps in R [16,17].

## Results

### Studies and records identified

**Figure 1:**
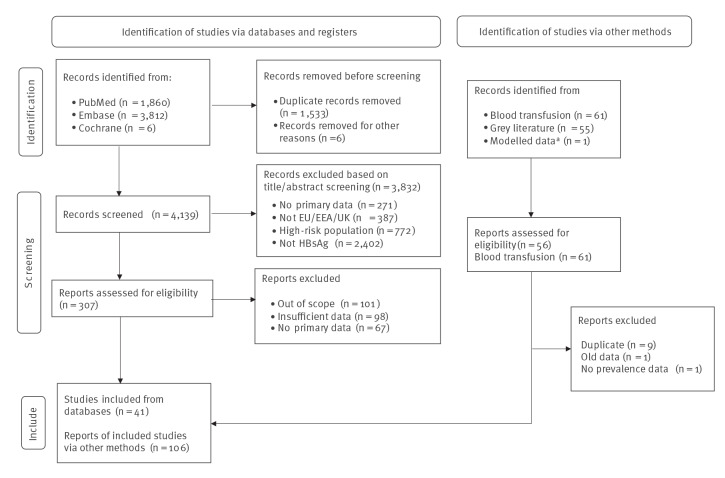
Prisma flow diagram for the systematic review of hepatitis B virus prevalence in the general population and key populations, EU/EEA/UK, 2018–2021

EEA: European Economic Area; EU: European Union; HBsAg: hepatitis B virus surface antigen; UK: United Kingdom.

^a^ Grey literature reporting modelled estimates for countries were included only if no other data were available.

A total of 5,678 articles were identified in the search update, with 4,139 accepted for screening after de-duplication and 3,832 articles excluded after review (Figure 1). After full-text screening of 307 studies, 41 met the inclusion criteria (Figure 1). We identified studies in five of the six included population groups, encompassing the general population (n = 12), migrant populations (n = 17), pregnant women (n = 5), MSM (n = 3) and people living in prisons (n = 4). No studies were identified for FTB. Additional data obtained from unpublished records and grey literature yielded a total of 106 records including 61 among FTB, nine in the general population, seven migrant population estimates, 16 in pregnant women, five among MSM and eight among people in prison. Supplementary Table S20 provides a detailed list of the number of identified estimates for the prevalence of chronic hepatitis B from 2018 to 2021 by country and population group. Additional estimates were provided from 12 countries for the general population (see the supplementary material for other studies). Additional studies obtained from unpublished and grey literature were included from 18 of 31 countries surveyed, across all population groups.

### First-time blood donors

**Figure 2:**
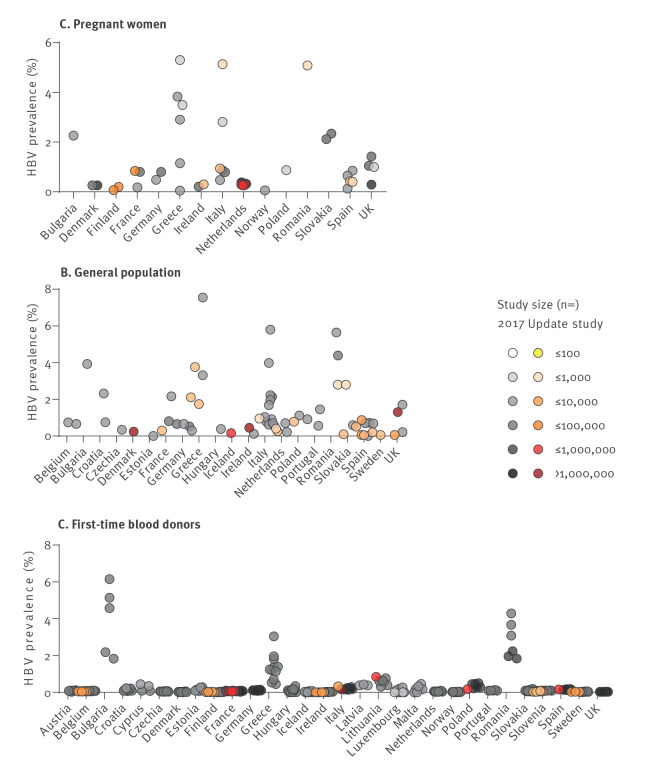
Hepatitis B virus prevalence estimates in sentinel populations, EU/EEA/UK, for studies published in 2005–2021 (n = 439 studies)

EEA: European Economic Area; EU: European Union; HBV: hepatitis B virus; UK: United Kingdom.

Included studies on FTB comprised 41.5% (n = 61) of the total number of new prevalence estimates in the update, covering data from 12 countries in the EU/EEA/UK. The FTB had a low HBV prevalence, with all estimates < 1%. The range of estimates varied from 0.0% in France to 0.8% in Lithuania (Figure 2).

Previous estimates from the 2017 database [11] are shown in grey scale, with updated estimates from this review represented in colour. Countries with no data are not shown. The update added 21 of 53 studies in pregnant women, 21 of 66 studies in the general population and 61 of 320 studies in first-time blood donors. A complete list of studies and prevalence estimates added in this study is provided in Supplementary Table S21.

### General population

Twenty-one new estimates of HBV prevalence in the general population were identified in this current review, covering 12 countries (Figure 2). Studies of prevalence estimates from three new countries (Denmark, Iceland and Sweden) with no previous estimate [7] among the general population were identified in this review. Note that the Danish general population estimate was derived from a model study [18].

Updated results for the general population ranged from 0.1% HBV prevalence in Spain [19] and the UK [20] to 2.8% in Slovakia [21] and 3.8% in Greece [22]. After excluding 13 studies with a high risk of bias, the highest prevalence estimate in the region was 1.7% in Greece [23]. Regarding the risk of bias, general population studies assessed as having low risk of bias (risk of bias score ≥ 4) were retrieved from France, Sweden, Greece, the Netherlands, Poland, Spain and the UK; these are listed in Supplementary Table S15.

### Pregnant women

Twenty-one studies (of the total update of 147 studies) among pregnant women were obtained from seven countries; see Supplementary Table S21 for the list of studies. The prevalence of HBV among pregnant women (Figure 2) ranged from 0.1% in Finland (unpublished) to 5.1% in Romania [24] and Italy [25]. In this population group, five studies were deemed to have low risk of bias (risk of bias score ≥ 2), more detail is found in Supplementary Table S17. These studies were from France, Ireland, the Netherlands, Romania and Spain. Prevalence estimates from studies with low risk of bias ranged from 0.3% in the Netherlands [26] to 0.8% in France [27]. The number of studies identified per country varied, with the Netherlands reporting many studies (n = 12) compared with one or two studies per country from Finland, France, Ireland, Italy, Romania and Spain (Figure 2). Eleven of the 12 prevalence estimates from The Netherlands contained routine annual antenatal screening programme data, which were collated between 2016 and 2021.

### Migrants

**Figure 3:**
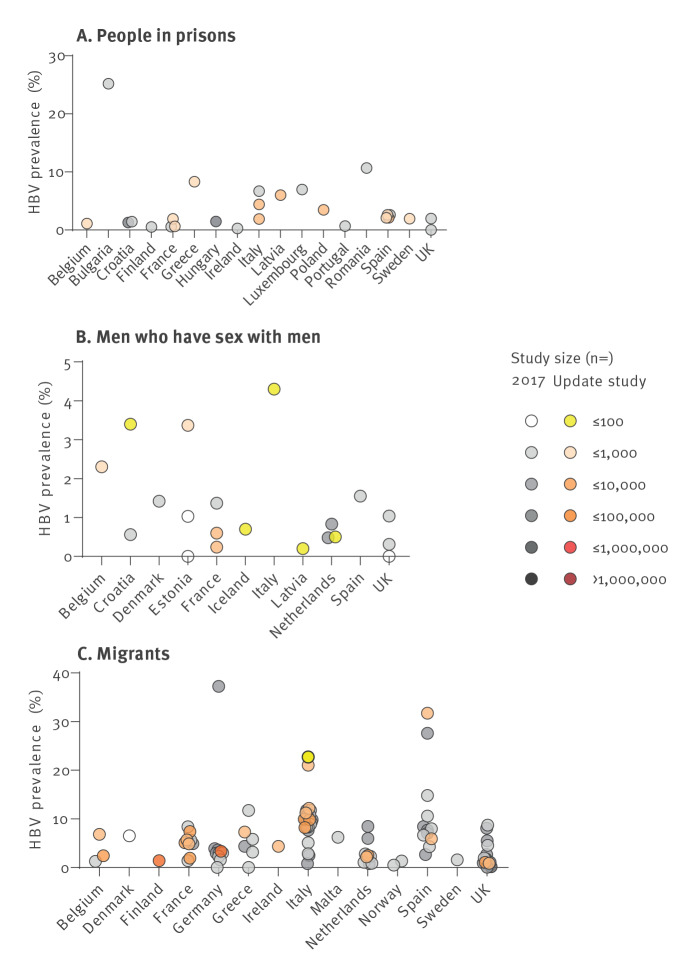
Hepatitis B virus prevalence estimates in key risk populations, including people in prison, men who have sex with men and migrants, EU/EEA/UK, 2005–2021 (n = 140 studies)

EEA: European Economic Area; EU: European Union; HBV: hepatitis B virus; MSM: men who have sex with men; UK: United Kingdom.

Studies among migrants were identified from 11 countries. Migrant populations mostly originated from Africa, Asia and Eastern Europe. Updated estimates among migrant populations that were assessed to have a low risk of bias (risk of bias score ≥ 1) ranged from 0.9% in the UK [28] to 31.7% in Spain [29] and are listed in Supplementary Table S16. Studies from two additional countries without data in the 2017 database were identified in this update (Finland: 1.4% [30], Ireland: 3.6% [31]) (Figure 3).

Previous estimates from the 2017 database are shown in grey scale, with updated estimates from this review represented in colour. The update added 12 of 26 studies in people in prison, eight of 19 studies in MSM and 24 of 95 studies in migrants. A complete list of studies and prevalence estimates added in this study is provided in Supplementary Table S21.

### Men who have sex with men

Among MSM, we identified eight data points from seven countries, with estimates for four new countries (Belgium, Iceland and Latvia). Results ranged from 0.2% HBV prevalence in France [32] to 3.4% in Croatia [33] (Figure 3). Studies with low risk of bias ranged from 0.2% [32] in France to 3.4% in Croatia [33] and Estonia (personal communication: K Rüütel, 4 May 2021).

### People in prisons

Twelve studies reporting estimates for people in prison were included from eight countries. The prevalence of HBV among people in prison (Figure 3) ranged from 0.6% in France [34] to 8.3% in Greece (personal communication: G Nikolopoulou, 17 May 2021). Prevalence estimates from studies with low risk of bias (risk of bias score ≥ 3, further details on the risk of bias scores are given in Supplementary Table S19) ranged from 1.1% in Belgium [35] to 8.3% in Greece (personal communication: G Nikolopoulou, 17 May 2021).

### Median prevalence

We compared, for each population group, the median prevalence among newly identified studies with those in the existing 2017 database [11] (Table). The median prevalence of HBsAg in the general population was lower (0.5%) than in the preceding review where it was estimated to be 0.7%, while it remained stable among FTB. Prevalence estimates among pregnant women decreased by 0.5% from the 0.8% estimated from the previous review. The median prevalence among MSM declined by 0.1% in the updated estimates. Among people in prison and migrants, the median prevalences increased by 0.6% and 0.9%, respectively.

**Table:** Median hepatitis B virus prevalence among studies identified in the updated review and existing 2017 database, EU/EEA/UK, 2005–2021 (n = 579)

Population group	% Median (IQR) Post-2017 database	% Median (IQR) 2017 database
First time blood donors	0.1 (0.0–0.1)	0.1 (0.0–0.2)
General population	0.5 (0.2–0.9)	0.7 (0.3–1.8)
Pregnant women	0.3 (0.3–0.4)	0.8 (0.3–1.6)
Men who have sex with men	0.7 (0.4–2.6)	0.8 (0.4–1.2)
People in prison	2.1 (1.9–3.7)	1.5 (0.6–5.7)
Migrants	5.8 (2.5–9.9)	4.9 (1.9–8.4)

EEA: European Economic Area; EU: European Union; IQR: interquartile range; UK: United Kingdom.

### Regional hepatitis B virus prevalence

**Figure 4:**
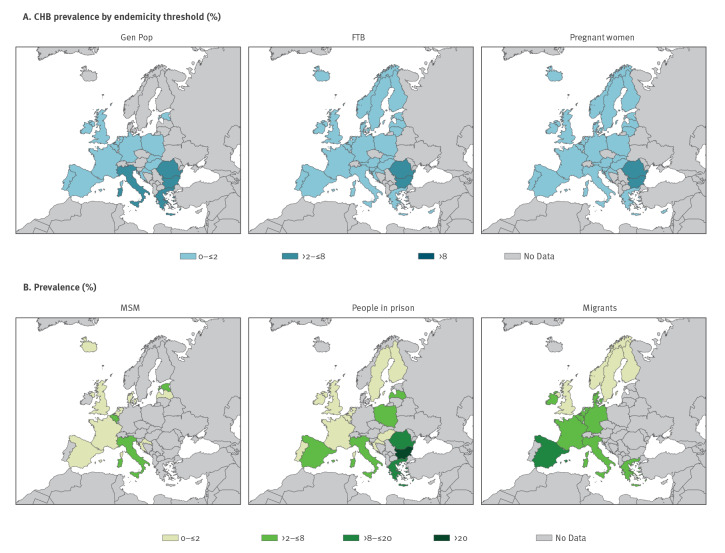
Weighted average prevalence estimates in different population groups, by country, EU/EEA/UK, 2005–2021 (n = 486)

CHB: chronic hepatitis B; EEA: European Economic Area; EU: European Union; FTB: first-time blood donors; GenPop: general population; MSM: men who have sex with men; UK: United Kingdom.

Weighted CHB prevalence estimates with low risk of bias from the total database indicated a low HBV prevalence (≤ 2%) among the general population, FTB and pregnant women in all regions with exceptions in Bulgaria, Greece, Italy and Romania (Figure 4) where prevalence ranged from 2.1% to 7.6%. The HBV prevalence among MSM was generally comparable to the general population (≤ 2%), except in Belgium, Croatia and Estonia (range: 2.3–3.4%). Estimates among migrants were typically higher in southern European regions (2.1–37.3%). In addition, prevalence among people in prison varied (2.1–25%), with 14 countries lacking data.

Our total results (2017 and post-2017 database) with low risk of bias were used to generate prevalence estimates displayed in these maps. Chronic HBV prevalence estimates are distinguished by the colour intensity in the legend.

## Discussion

Updated estimates identifying an additional 147 HBsAg prevalence estimates across the EU/EEA/UK region suggest that < 2% of the general population have CHB. The number of high-quality studies in each population group was proportional to the 2017 database. However, we noted some changes over time, with a reduction in prevalence for most countries that reported new estimates. These reductions could reflect the impact of global vaccination and improved prevention and control strategies but could also relate to methodological differences in recruitment, sampling and representativeness. A recent progress report indicated that, in the period 2016 to 2019, Italy and the Netherlands achieved the elimination targets and that Croatia and the UK were on the verge of achieving this status [36].

Even though 40% of countries provided additional data on key risk groups from grey literature or unpublished sources, studies covering key risk populations were insufficient, in particular for eastern Europe, where data were often lacking and sample sizes were small. The highest HBV prevalences were reported among people in prison (8.3%) and migrants (31.7%) – with higher estimates for these populations more common in southern and eastern countries. 

Our updated estimate for HBV prevalence among FTB was < 2%, similar to estimates found in the previous review done by ECDC [7]. Recent serosurveillance data from a third of the in-country focal points showed similar estimates, ranging from 0.0% in Ireland to 0.8% in Lithuania. Updated data were lacking from Bulgaria, Greece and Romania where the prevalence was reported to be much higher (> 2%) in the previous study [7]. The previous systematic review had a longer inclusion period spanning from 2005 to 2015, which may explain the wider variation of estimates. A further factor that can affect the prevalence among donors is that there is usually no payment for donations, and most countries have strict pre-screening in place, both of which limit people with risk factors donating blood. The median prevalence trend among FTB was stable when comparing the 2017 and post-2017 database.

Among pregnant women, HBV prevalence was lower than in the previous review by 0.5%, which may reflect progressive vaccination coverage and the ageing of the vaccinated cohorts [37]. Prevalence among pregnant women is low but the prevention of mother-to-child transmission (MTCT) is important as babies infected through this route are at high risk for developing CHB without intervention. Knowledge of maternal vaccination status would allow a targeted approach in groups with higher risk. Vertical transmission remains an important source of chronic infections in the EU/EEA/UK, particularly among first- and second-generation migrants, relating to infections that occurred in their countries of origin [6]. Countries should ensure comprehensive strategies to reduce risk of MTCT, including universal birth-dose vaccination and antenatal screening combined with post-exposure prophylaxis for infants born to infected mothers. Surveillance data in our database indicate that migrant populations are disproportionately affected by HBV, so antenatal programmes need to be sensitive and adapted to the needs of different migrant groups.

Pregnant women and FTB can be used as proxy populations for estimating HBV prevalence in the general population. Nonetheless, there are discrepancies between these groups, and pregnant women tend to have higher prevalence estimates than FTB. Both population groups have inherent biases such as self-selection and screening before blood donation [12]. Pregnant women are considered not fully representative of the general population because of age and gender biases. In addition, migrant populations may be overrepresented, which may overestimate prevalence among pregnant women [12].

Among the majority of studies on MSM, HBV prevalence is broadly similar to the general population. Assuming most MSM are infected as adults via sexual activity, the majority of those infected should clear their acute infection. Previous work did not categorise MSM as a high-risk group (> 2%), however, it noted that caution should be taken because of the wide variation of HBV estimates [13]. There were few data among MSM compared with other key risk groups, and studies were notably missing in eastern Europe. Belgium, Croatia and Estonia seemed to have higher HBV prevalence (2.3–3.4%) within their MSM populations than other countries with available data. Typically, MSM are offered HBV vaccination through targeted interventions, and that this may be contributing to the low prevalence estimates. In the 2017 European MSM Internet Survey (EMIS-2017), it was reported that in the EU/EEA, 44.8% of MSM had been vaccinated against hepatitis B [38]. Estonia had a low vaccination coverage (28.9%) among its MSM population, which may explain the higher prevalence observed in this country [38]. Given the small sample sizes for these estimates, the observed higher prevalence may not be representative of MSM populations in some countries. Additionally, data were often collected from volunteers attending sexually transmitted infections clinics or community centres, and thus may not be representative samples.

Many migrants entering into Europe originate from regions where HBV is endemic, resulting in a prevalence higher than the general population [39,40]. Accordingly, our study found high prevalence estimates in these populations. Italy and Spain recorded some of the highest HBV prevalence among migrants (32% and 21%, respectively) whereas in the last review, the prevalence was highest in Germany with estimates reaching 37% [7]. These were newly arrived migrants (≤ 12 months after entry in the country) and predominantly from sub-Saharan Africa. Conversely, most eastern European countries seemed to have no records of HBV and migration status.

Our data among migrant populations were based on specific ethnic groups, that are varied and diverse with wide-ranging HBV prevalences. This highlights the importance of determining the type of population sampled, as HBV prevalence is higher among refugees and asylum seekers compared with other types of migrants (economic migrants, students etc.) [15,41]. This may reflect the ‘healthy migrant effect’, which alludes to younger and healthier economic migrants relocating for professional reasons or differences in the level of HBV in the countries of birth of different migrant groups [40].

The prevalence of HBV among people in prison could be influenced by multiple factors that render them vulnerable to HBV infection. Injecting drug use is more common among the imprisoned population, there is more frequent use of unsterile tattooing equipment [42] and vaccination programmes are suboptimal [43]. Previous studies have indicated that migrants may be overrepresented in the prison population [13]. Evidence from our update shows that people in prison in Bulgaria, Poland and Romania have high HBV prevalence. Bulgaria and Romania have consistently high HBV prevalence, and the added vulnerability of being in prison may explain the exceptionally high prevalence (> 10%) in their prison populations. Needle and syringe programmes in prisons could reduce transmission [43]. Crucially, a robust screening and treatment strategy should be implemented in these populations [13]. 

The observed median prevalence in people in prison and migrants increased in our update, and invariably remained higher than in the general population. These groups are underrepresented in general population or FTB surveys. In view of this, reports of national estimates of prevalence in the population as a whole should be adjusted by population size estimates of the migrant, prison and PWID populations in the countries and their HBV prevalence. Screening and linkage to care data are lacking widely across the region in all population groups, with a previous ECDC survey reporting data for just 12 of 31 countries and finding that eight of these countries did not meet the target of diagnosing 50% of people living with HBV [44]. Further evaluation of this in more populations would be informative for understanding where there are gaps in the cascade of care. 

A key limitation of the review is the variable quality of studies identified. Several studies had limitations in terms of sample size, sampling method and population sampled, which affected the generalisability of results. Ideally, we could have modelled HBV prevalence combining sentinel and risk population data, but this was not possible without additional evidence on the size of key risk populations within countries and those data was not available. The range of results seen across countries could be attributed to the variable quality of studies and sample sizes, therefore, there is a lot more uncertainty in the estimates. Data on MSM are typically lacking in countries where MSM are more stigmatised. The variations seen across the EU/EEA/UK could be due to the diversity of transmission routes and implementation of prevention and control strategies [13]. There has possibly been more migration into Greece, Italy and Spain from high-endemic areas in recent years [45]. In the 2017 database, data from FTB were obtained from the European Directorate for the Quality of Medicines and HealthCare (EDQM) of the council of Europe. Our update only included countries that provided recent blood donor data, and these were collected from in-country experts’ responses to our questionnaire. The quality assessment of migrant data was an additional component in our update that was not undertaken in the existing database. These changes limit a point-by-point comparison between the existing database and our update.

Additional methodological differences in study design and data collection and pooling of prevalence estimates to calculate weighted mean prevalence should be conditionally interpreted, especially when deducing the prevalence in the general population from proxy populations. Previous work has cautioned against using sentinel groups as a proxy for the general population because of the diversity between groups [12]. Furthermore, FTB may not accurately represent the general population because of selection bias [12]. For consistency and better interpretation of results, future analysis could assess the quality of migrant data in the existing database; a critical appraisal of each study (including region of origin and type of screening) would help understand why estimates vary and would identify higher quality studies which will provide a closer representation of the migrant group.

## Conclusions

We found new estimates of HBsAg prevalence from eight countries that had no previous records for some population groups. Comparing the median prevalence in the existing database and this update, the characteristic higher HBV prevalence among key risk groups remained high, with elevated prevalence trends among migrants and people in prison. The prevalence among other groups was either unchanged or decreased. Improved strategies are required to identify individuals infected with HBV, and data from this study can be used to highlight key populations where increased efforts around testing and linkage to care are most needed to address current health gaps. Our recommendation is that HBV screening and treatment should be targeted to people in prison and migrants. Strategies to increase the numbers of HBV cases identified will also provide opportunities to link more people living with HBV into the continuum of care. This would subsequently limit transmission, morbidity and mortality. In addition, vaccination policies are inadequate if they focus solely on infants and are not extended to cover all populations at higher risk of infection who may not have been vaccinated as a child. Targeted interventions in key populations with the highest prevalence are required to accelerate progress towards eliminating hepatitis B in the EU/EEA and the UK.

## Supplementary Data

628000 Supplement
