# Supplementary material for: Estimates of hepatitis B virus prevalence among general population and key risk groups in EU/EEA/UK countries: a systematic review
Source: Euro Surveill. 2023 Jul 27;28(30):2200738. doi: 10.2807/1560-7917.ES.2023.28.30.2200738 (PMC10375838; doi:10.2807/1560-7917.ES.2023.28.30.2200738)
Supplement: Supplement [file 22-00738_BIVEGETE_Supplement.pdf]

Supplement

Estimates of hepatitis B virus prevalence among general population and key risk groups in EU/EEA/UK countries: a systematic review.

Sandra Bivegete , Anna L McNaughton, Adam Trickey, Zak Thornton, Becky Scanlan, Aaron G Lim, Lina Nerlander, Hannah Fraser, Josephine G Walker, Matthew Hickman, Peter Vickerman, Helen Johnson, Erika Duffell, Ellen Brooks-Pollock, Hannah Christensen,

This supplementary material is hosted by Eurosurveillance as supporting information alongside the article [Estimates of hepatitis B virus prevalence among general population and key risk groups in EU/EEA/UK countries: a systematic review], on behalf of the authors, who remain responsible for the accuracy and appropriateness of the content. The same standards for ethics, copyright, attributions and permissions as for the article apply. Supplements are not edited by Eurosurveillance and the journal is not responsible for the maintenance of any links or email addresses provided therein.

Table of contents

Table S1: Example of PubMed Search Results 1
Table S2: Example of Embase Search Results 6
Table S3: Example of Cochrane Search Results 7
Table S4: Framework for assessing risk of bias (general population) 7
Table S5: Framework for assessing risk of bias: pregnant women 8
Table S6: Framework for assessing risk of bias (men who have sex with men) 8
Table S7: Framework for assessing risk of bias (people in prison) 8
Table S8: Framework for assessing risk of bias (migrants) 8
Table S9: Weighted HBV prevalence of studies among pregnant women in the EU/EEA and UK 10
Table S10: Weighted HBV prevalence of identified studies among first-time blood donors in the EU/EEA and UK 10
Table S11: Weighted HBV prevalence of identified studies in the general population in the EU/EEA and UK 11
Table S12: Weighted HBV prevalence of identified studies among migrants in the EU/EEA and UK 12
Table S13: Weighted HBV prevalence of identified studies among men who have sex with men in the EU/EEA 12
Table S14: Weighted HBV prevalence of identified studies among people in prison in the EU/EEA and UK 12
Table S15: Results of the risk of bias assessment for the general population 13
Table S16: Results of the risk assessment for migrants 14
Table S17: Results of the risk assessment for pregnant women 15
Table S18: Results of the risk assessment for men who sex with men 16
Table S19: Results of the risk assessment for People in prison 16
Table S20: Number of identified estimates for the prevalence of chronic hepatitis B (HBsAg) from 2018-2021 17
Table S21: Sample size of studies presented in figure 2 and 3. 18

Table S1-Table S3 are extracted sections from the full search, including filters and limits applied.

Table S1: Example of PubMed Search Results

Search number	Query	Search Details	Search Hits
4	("European Union"[Mesh] OR "Europe"[Mesh:noexp] OR Europe*[tw] OR Europa*[tw] OR EU[tw] OR EEA[tw] OR EFTA[tw] OR "EU/EEA"[tw] OR "EU/EFTA"[tw] OR ECSC[tw] OR Euratom[tw] OR Eurozone[tw] OR EEC[tw] OR ec[tw] OR (Schengen[tw] AND (area[tw] OR countr*[tw] OR region*[tw] OR state[tw] OR states[tw])) OR Euroregion[tw] OR Euroregions[tw] OR "Europe, Eastern"[Mesh:noexp] OR "Balkan Peninsula"[Mesh] OR Balkan[tw] OR Balkans[tw] OR "Baltic States"[Mesh] OR Baltic[tw] OR "Mediterranean Region"[Mesh] OR (Mediterranean[tw] AND (area[tw] OR countr*[tw] OR region*[tw] OR state[tw] OR states[tw])) OR (Alpine[tw] AND (area[tw] OR countr*[tw] OR region*[tw] OR state[tw] OR states[tw])) OR "Scandinavian and Nordic Countries"[Mesh] OR Scandinavia[tw] OR Scandinavian[tw] OR "Nordic country"[tw] OR "Nordic countries"[tw] OR "Nordic state"[tw] OR "Nordic states"[tw] OR Danubian[tw] OR "Iberian peninsula"[tw] OR "Peninsula iberica"[tw] OR "Peninsule Iberique"[tw] OR "Iberiar Penintsula"[tw] OR Iberia[tw] OR Anatolia[tw] OR Anadolu[tw] OR Anatole[tw] OR Anatolian[tw] OR "Yugoslavia"[Mesh] OR Yugoslavia[tw] OR "Czechoslovakia"[Mesh] OR Czechoslovakia[tw] OR "Czecho Slovakia"[tw] OR Ceskoslovensko[tw] OR "Cesko slovensko"[tw] OR Benelux[tw] OR Fennoscandia[tw] OR "Fenno Scandinavia"[tw] OR Fennoskandi*[tw] OR (Visegrad[tw] AND (Group[tw] OR Four[tw] OR Triangle[tw])) OR "Visegradska ctyrka"[tw] OR "Visegradska skupina"[tw] OR "Visegradi Egyuttmukodes"[tw] OR "Visegradi	"European Union"[MeSH Terms] OR "Europe"[MeSH Terms:noexp] OR "europe*"[Text Word] OR "europa*"[Text Word] OR "EU"[Text Word] OR "EEA"[Text Word] OR "EFTA"[Text Word] OR "EU/EEA"[Text Word] OR "EU/EFTA"[Text Word] OR "ECSC"[Text Word] OR "Euratom"[Text Word] OR "Eurozone"[Text Word] OR "EEC"[Text Word] OR "ec"[Text Word] OR ("Schengen"[Text Word] AND ("area"[Text Word] OR "countr*"[Text Word] OR "region*"[Text Word] OR "state"[Text Word] OR "states"[Text Word])) OR "Euroregion"[Text Word] OR "Euroregions"[Text Word] OR "europe, eastern"[MeSH Terms:noexp] OR "Balkan Peninsula"[MeSH Terms] OR "Balkan"[Text Word] OR "Balkans"[Text Word] OR "Baltic States"[MeSH Terms] OR "Baltic"[Text Word] OR "Mediterranean Region"[MeSH Terms] OR ("Mediterranean"[Text Word] AND ("area"[Text Word] OR "countr*"[Text Word] OR "region*"[Text Word] OR "state"[Text Word] OR "states"[Text Word])) OR ("Alpine"[Text Word] AND ("area"[Text Word] OR "countr*"[Text Word] OR "region*"[Text Word] OR "state"[Text Word] OR "states"[Text Word])) OR "Scandinavian and Nordic Countries"[MeSH Terms] OR "Scandinavia"[Text Word] OR "Scandinavian"[Text Word] OR "Nordic country"[Text Word] OR "Nordic countries"[Text Word] OR "Nordic states"[Text Word] OR "Danubian"[Text Word] OR "Iberian peninsula"[Text Word] OR "Peninsula iberica"[Text Word] OR "Peninsule Iberique"[Text Word] OR "Iberia"[Text Word] OR "Anatolia"[Text Word] OR "Anadolu"[Text Word] OR "Anatole"[Text Word] OR "Anatolian"[Text Word] OR "Yugoslavia"[MeSH Terms] OR	3579578

Search number	Query	Search Details	Search Hits
	negyek"[tw] OR "Grupa Wyszehradzka"[tw] OR "Vysehradska skupina"[tw] OR "Vysehradska stvorka"[tw]) OR ("Austria"[Mesh] OR Austria*[tw] OR Osterreich*[tw] OR Oesterreich*[tw]) OR ("Belgium"[Mesh] OR Belgi*[tw] OR Belge*[tw] OR Belg[tw]) OR ("Bulgaria"[Mesh] OR Bulgaria*[tw] OR Balgariya[tw] OR Balgarija[tw] OR Blagoevgrad*[tw]) OR ("Croatia"[Mesh] OR Croat*[tw] OR Hrvatsk*[tw] OR hrvati[tw]) OR ("Cyprus"[Mesh] OR Cyprus[tw] OR Cypriot*[tw] OR Kypros[tw] OR Kibris*[tw] OR kypriaki[tw] OR Kyprioi[tw]) OR ("Czech Republic"[Mesh] OR Czech*[tw] OR Cesky[tw] OR Ceska[tw] OR Cech[tw] OR Cestina[tw]) OR ("Denmark"[Mesh] OR Denmark[tw] OR Danish*[tw] OR dane[tw] OR danes[tw] OR Danmark[tw] OR dansk*[tw] OR Hovedstaden[tw]) OR ("Estonia"[Mesh] OR Estonia*[tw] OR Eesti[tw] OR Eestlased[tw] OR Eestlane[tw]) OR ("Finland"[Mesh] OR Finland[tw] OR Finnish*[tw] OR Finn[tw] OR Finns[tw] OR Suomi[tw] AND Suomen[tw] OR Suomalaiset[tw]) OR ("France"[Mesh] OR France[tw] OR French*[tw] OR Francais*[tw]) OR ("Germany"[Mesh] OR German*[tw] OR Deutsch*[tw] OR Bundesrepublik[tw]) OR ("Greece"[Mesh] OR Greece[tw] OR "Hellenic republic"[tw] OR Greek*[tw]) OR ("Hungary"[Mesh] OR Hungar*[tw] OR Magyarorszag[tw] OR Magyar*[tw]) OR ("Ireland"[Mesh] OR Ireland[tw] OR Eire[tw] OR Irish*[tw]) OR ("Italy"[Mesh] OR Italy[tw] OR Italia*[tw]) OR ("Latvia"[Mesh] OR Latvi*[tw]) OR ("Lithuania"[Mesh] OR Lithuania*[tw] OR "Lietuvos Respublika"[tw] OR Lietuva[tw] OR	"Yugoslavia"[Text Word] OR "Czechoslovakia"[MeSH Terms] OR "Czechoslovakia"[Text Word] OR "Czecho Slovakia"[Text Word] OR "Benelux"[Text Word] OR "Fennoscandia"[Text Word] OR "Fenno Scandinavia"[Text Word] OR ("Visegrad"[Text Word] AND ("Group"[Text Word] OR "Four"[Text Word] OR "Triangle"[Text Word])) OR ("Austria"[MeSH Terms] OR "austria*"[Text Word] OR "osterreich*"[Text Word] OR "oesterreich*"[Text Word]) OR ("Belgium"[MeSH Terms] OR "belgi*"[Text Word] OR "belge*"[Text Word] OR "Belg"[Text Word]) OR ("Bulgaria"[MeSH Terms] OR "bulgaria*"[Text Word] OR "blagoevgrad*"[Text Word]) OR ("Croatia"[MeSH Terms] OR "croat*"[Text Word] OR "hrvatsk*"[Text Word]) OR ("Cyprus"[MeSH Terms] OR "Cyprus"[Text Word] OR "cypriot*"[Text Word] OR "Kypros"[Text Word]) OR ("Czech Republic"[MeSH Terms] OR "czech*"[Text Word] OR "Cesky"[Text Word] OR "Ceska"[Text Word] OR "Cech"[Text Word]) OR ("Denmark"[MeSH Terms] OR "Denmark"[Text Word] OR "danish*"[Text Word] OR "dane"[Text Word] OR "danes"[Text Word] OR "Danmark"[Text Word] OR "dansk*"[Text Word] OR "Hovedstaden"[Text Word]) OR ("Estonia"[MeSH Terms] OR "estonia*"[Text Word] OR "Eesti"[Text Word]) OR (("Finland"[MeSH Terms] OR "Finland"[Text Word] OR "finnish*"[Text Word] OR "Finn"[Text Word] OR "Finns"[Text Word] OR "Suomi"[Text Word]) AND "Suomen"[Text Word]) OR ("France"[MeSH Terms] OR "France"[Text Word] OR "french*"[Text Word] OR "francais*"[Text Word]) OR ("Germany"[MeSH Terms] OR	

Search number	Query	Search Details	Search Hits
	lietuviu[tw]) OR ("Luxembourg"[Mesh] OR Luxembourg*[tw] OR Luxemburg[tw] OR Letzebuerg[tw]) OR ("Malta"[Mesh] OR Malta[tw] OR Maltese*[tw] OR Maltin[tw]) OR ("Poland"[Mesh] OR Poland[tw] OR Polska[tw] OR Polish[tw] OR Pole[tw] OR Poles[tw] OR Polski[tw] OR Polak[tw] OR Polka[tw] OR Polacy[tw]) OR ("Portugal"[Mesh] OR Portugal[tw] OR Portugues*[tw]) OR ("Romania"[Mesh] OR Romania*[tw] OR Rumania*[tw] OR Roumania*[tw] OR Romani[tw] OR Rumani[tw]) OR ("Slovakia"[Mesh] OR Slovakia[tw] OR Slovensk*[tw] OR Slovak*[tw] OR Slovaci[tw] OR Slovenki[tw]) OR ("Slovenia"[Mesh] OR Slovenia*[tw] OR Slovenija[tw] OR slovensk*[tw] OR Slovenci[tw] OR Slovene*[tw]) OR ("Spain"[Mesh] OR Spain[tw] OR Espana[tw] OR Spanish[tw] OR Espanol*[tw] OR Spaniard*[tw]) OR ("Sweden"[Mesh] OR Sweden[tw] OR Sverige[tw] OR Swedish[tw] OR Svenska[tw] OR svenskar[tw] OR Swede[tw] OR Swedes[tw]) OR ("Great Britain"[Mesh] OR GB[tw] OR "United kingdom"[tw] OR UK[tw] OR Britain[tw] OR British[tw] OR England[tw] OR English[tw] OR Scotland[tw] OR Scottish[tw] OR Scots[tw] OR Wales[tw] OR Cymru[tw] OR Welsh[tw] OR "North Ireland"[tw] OR "Northern Ireland"[tw] OR Irish[tw])	"german*"[Text Word] OR "deutsch*"[Text Word] OR "Bundesrepublik"[Text Word]) OR ("Greece"[MeSH Terms] OR "Greece"[Text Word] OR "Hellenic republic"[Text Word] OR "greek*"[Text Word]) OR ("Hungary"[MeSH Terms] OR "hungar*"[Text Word] OR "Magyarorszag"[Text Word] OR "magyar*"[Text Word]) OR ("Ireland"[MeSH Terms] OR "Ireland"[Text Word] OR "Eire"[Text Word] OR "irish*"[Text Word]) OR ("Italy"[MeSH Terms] OR "Italy"[Text Word] OR "italia*"[Text Word]) OR ("Latvia"[MeSH Terms] OR "latvi*"[Text Word]) OR ("Lithuania"[MeSH Terms] OR "lithuania*"[Text Word]) OR ("Luxembourg"[MeSH Terms] OR "luxembourg*"[Text Word] OR "Luxemburg"[Text Word] OR "Letzebuerg"[Text Word]) OR ("Malta"[MeSH Terms] OR "Malta"[Text Word] OR "maltese*"[Text Word] OR "Maltin"[Text Word]) OR ("Poland"[MeSH Terms] OR "Poland"[Text Word] OR "Polska"[Text Word] OR "Polish"[Text Word] OR "Pole"[Text Word] OR "Poles"[Text Word] OR "Polski"[Text Word] OR "Polak"[Text Word] OR "Polka"[Text Word]) OR ("Portugal"[MeSH Terms] OR "Portugal"[Text Word] OR "portugues*"[Text Word]) OR ("Romania"[MeSH Terms] OR "romania*"[Text Word] OR "rumania*"[Text Word] OR "roumania*"[Text Word] OR "Romani"[Text Word] OR "Rumani"[Text Word]) OR ("Slovakia"[MeSH Terms] OR "Slovakia"[Text Word] OR "slovensk*"[Text Word] OR "slovak*"[Text Word]) OR ("Slovenia"[MeSH Terms] OR "slovenia*"[Text Word] OR "Slovenija"[Text Word] OR "slovensk*"[Text Word] OR	

Search number	Query	Search Details	Search Hits
		"slovene*"[Text Word]) OR ("Spain"[MeSH Terms] OR "Spain"[Text Word] OR "Espana"[Text Word] OR "Spanish"[Text Word] OR "espanol*"[Text Word] OR "spaniard*"[Text Word]) OR ("Sweden"[MeSH Terms] OR "Sweden"[Text Word] OR "Sverige"[Text Word] OR "Swedish"[Text Word] OR "Svenska"[Text Word] OR "Swede"[Text Word] OR "Swedes"[Text Word]) OR ("GB"[Text Word] OR "United kingdom"[Text Word] OR "UK"[Text Word] OR "Britain"[Text Word] OR "British"[Text Word] OR "England"[Text Word] OR "English"[Text Word] OR "Scotland"[Text Word] OR "Scottish"[Text Word] OR "Scots"[Text Word] OR "Wales"[Text Word] OR "Cymru"[Text Word] OR "Welsh"[Text Word] OR "North Ireland"[Text Word] OR "Northern Ireland"[Text Word] OR "Irish"[Text Word])	
3	#1 AND #2	("Prevalence"[MeSH Terms] OR "prevalence*"[Title/Abstract] OR "Population Surveillance"[MeSH Terms] OR "Seroepidemiologic Studies"[MeSH Terms:noexp] OR "seroprevalence*"[Title/Abstract] OR "seroepidemiolog*"[Title/Abstract] OR "sero epidemiologic"[Title/Abstract] OR "sero epidemiological"[Title/Abstract] OR "sero epidemiology"[Title/Abstract] OR "serosurvey*"[Title/Abstract] OR "serolog*"[Title] OR "epidemiolog*"[Title] OR "surveillance"[Title]) AND ("hepatitis B"[MeSH Terms] OR "hepatitis c"[MeSH Terms] OR "Hepatitis B virus"[MeSH Terms] OR "hepacivirus"[MeSH Terms] OR "hepatitis B"[Title/Abstract] OR "hepatitis c"[Title/Abstract] OR "hepaciviru*"[Title/Abstract] OR "hbv"[Title/Abstract] OR	27099

Search number	Query	Search Details	Search Hits
		"hcv"[Title/Abstract] OR "hbsag"[Title/Abstract] OR "hbs ag"[Title/Abstract] OR "hepatitis b surface antigens"[MeSH Terms] OR "Australia Antigen"[Title/Abstract] OR "Australia Antigens"[Title/Abstract] OR "hepatitis c antibodies"[MeSH Terms] OR "Hepatitis C Antigens"[MeSH Terms] OR "Hepatitis B Antibodies"[MeSH Terms])	
2	"hepatitis B"[MeSH Terms] OR "hepatitis c"[MeSH Terms] OR "Hepatitis B virus"[Mesh] OR "hepacivirus"[Mesh] OR "hepatitis b"[TIAB] OR "hepatitis c"[TIAB] OR hepaciviru*[TIAB] OR "hbv"[TIAB] OR "hcv"[TIAB] OR "hbsag"[TIAB] OR "hbs ag"[TIAB] OR "hepatitis b surface antigens"[MeSH Terms] OR "Australia Antigen"[TIAB] OR "Australia Antigens"[TIAB] OR "hepatitis c antibodies"[MeSH Terms] OR "Hepatitis C Antigens"[Mesh] OR "Hepatitis B Antibodies"[Mesh]	"hepatitis B"[MeSH Terms] OR "hepatitis c"[MeSH Terms] OR "Hepatitis B virus"[MeSH Terms] OR "hepacivirus"[MeSH Terms] OR "hepatitis B"[Title/Abstract] OR "hepatitis c"[Title/Abstract] OR "hepaciviru*"[Title/Abstract] OR "hbv"[Title/Abstract] OR "hcv"[Title/Abstract] OR "hbsag"[Title/Abstract] OR "hbs ag"[Title/Abstract] OR "hepatitis b surface antigens"[MeSH Terms] OR "Australia Antigen"[Title/Abstract] OR "Australia Antigens"[Title/Abstract] OR "hepatitis c antibodies"[MeSH Terms] OR "Hepatitis C Antigens"[MeSH Terms] OR "Hepatitis B Antibodies"[MeSH Terms]	181102
1	"Prevalence"[Mesh] OR Prevalence*[TIAB] OR "Population Surveillance"[Mesh] OR "Seroepidemiologic Studies"[Mesh:NoExp] OR seroprevalence*[TIAB] OR seroepidemiolog*[TIAB] OR "sero epidemiologic"[TIAB] OR "sero epidemiological"[TIAB] OR "sero epidemiology"[TIAB] OR serosurvey*[TIAB] OR serolog*[TI] OR epidemiolog*[TI] OR surveillance[TI]	"Prevalence"[MeSH Terms] OR "prevalence*"[Title/Abstract] OR "Population Surveillance"[MeSH Terms] OR "Seroepidemiologic Studies"[MeSH Terms:noexp] OR "seroprevalence*"[Title/Abstract] OR "seroepidemiolog*"[Title/Abstract] OR "sero epidemiologic"[Title/Abstract] OR "sero epidemiological"[Title/Abstract] OR "sero epidemiology"[Title/Abstract] OR "serosurvey*"[Title/Abstract] OR "serolog*"[Title] OR "epidemiolog*"[Title] OR "surveillance"[Title]	958104

Table S2: Example of Embase Search Results

No.	Query	Search Hits
#5	(epidemiolog* NEAR/5 ('hepatitis b' OR 'hepatitis c' OR hepaciviru* OR 'hbv' OR 'hcv' OR hbsag OR 'hbs ag' OR 'australia antigen' OR 'australia antigens')):ti	1530
#4	(prevalence NEAR/5 ('hepatitis b' OR 'hepatitis c' OR hepaciviru* OR 'hbv' OR 'hcv' OR hbsag OR 'hbs ag' OR 'australia antigen' OR 'australia antigens')):ab,ti	16724
#3	#1 AND #2	33335
#2	'hepatitis b'/exp OR 'hepatitis c'/exp OR 'hepatitis b virus'/exp OR 'hepatitis c virus'/exp OR 'hepatitis b':ab,ti OR 'hepatitis c':ab,ti OR hepaciviru*:ab,ti OR 'hbv':ab,ti OR 'hcv':ab,ti OR hbsag:ab,ti OR 'hbs ag':ab,ti OR 'hepatitis b antibody'/exp OR 'hepatitis b surface antigen'/exp OR 'australia antigen':ab,ti OR 'australia antigens':ab,ti OR 'hepatitis c antigen'/exp OR 'hepatitis c antibody'/exp	298586
#1	'prevalence'/exp OR 'seroepidemiology'/exp OR 'disease surveillance'/exp OR 'sero epidemiology':ab,ti OR 'sero epidemiological':ab,ti OR 'sero epidemiologic':ab,ti OR seroepidemiolog*:ab,ti OR surveillance:ti OR serolog*:ti OR serosurvey*:ab,ti OR seroprevalence*:ab,ti OR 'population surveillance':ab,ti	907924

Table S3: Example of Cochrane Search Results

Search Name:		Cochrane
**ID**		**Search Hits**
#1	MeSH descriptor: [Prevalence] explode all trees	4611
#2	MeSH descriptor: [Population Surveillance] explode all trees	513
#3	MeSH descriptor: [Seroepidemiologic Studies] 2 tree(s) exploded	119
#15	#6 or #7 or #8 or #9 or #10 or #11 or #12 or #13 or #14	18584
#16	#15 and #5 Publication Year from 2018 to 2021	6

Table S4: Framework for assessing risk of bias (general population)

Domain	Scores	Description
Age	0	Clear age bias (towards children for example), not representative for general population; no information on age.
	1	No clear bias in age profile of respondents; representative for general population age distribution
Gender	0	Clear bias in gender; no information to suggest representativeness for general population
	1	No clear bias in gender distribution of subjects; could be considered representative if information is limited
Sampling method	0	Non-random or non-exhaustive
	1	Exhaustive or random and<60% response rate or no info.
	2	Exhaustive or random and >60% response rate
Population coverage	0	Single centre/local
	1	Multi-centre/local or regional
	2	Multi-centre/national

Domains were scored and the total risk of bias was assessed by adding up all four domains. The highest score of 6 indicated the lowest risk of bias and 0 the highest risk [1].

Table S5: Framework for assessing risk of bias: pregnant women

Domain	Scores	Description
Sampling method	0	Non-random or non-exhaustive
	1	Exhaustive or random
Population coverage	0	Single centre/local
	1	Multi-centre/local or regional
	2	Multi-centre/national

Domains were scored and the total risk of bias was assessed by adding up all domains. The highest score of 3 indicated the lowest risk of bias and 0 the highest risk[1].

Table S6: Framework for assessing risk of bias (men who have sex with men)

Domain	Scores	Description
Population coverage	0	Single venue
	1	Multi-centre/single venue type
	2	Multi-centre/multi-venue type

The total risk of bias was assessed by adding up the domain. The highest score of 2 indicated the lowest risk of bias and 0 the highest risk [1].

Table S7: Framework for assessing risk of bias (people in prison)

Domain	Scores	Description
Age	0	Clear age bias (i.e., among juvenile offenders only); no information
	1	No clear bias in age profile of subjects
Gender	0	Clear bias in gender; no information
	1	No clear bias in gender distribution of subjects; could be considered representative if information is limited
% PWID	0	Exclusively among PWID/former PWID prisoners
	1	PWID not used to select subjects; no bias toward PWID
Sampling method	0	Non-random or non-exhaustive
	1	Exhaustive or random
	2	Exhaustive or random and >60% response rate
Population coverage	0	Single centre/local
	1	Multi-centre/local or regional
	2	Multi-centre/national

Domains were scored and the total risk of bias was assessed by adding up all four domains. The highest score of 6 indicated the lowest risk of bias and 0 the highest risk[1].

Table S8: Framework for assessing risk of bias (migrants)

Domain	Sample size	Description	Scores
A	Any	AND	1.2

Domain	Sample size	Description	Scores
		Nationally or regionally representative sample	
		Stratified, multistage, random sample	
		Design described and documented	
		Demographics of subjects reported (age and sex)	
		Potential for bias is limited	
B	Any	Immigrant study unless significant potential for bias (e.g., self-selection as in free screening program)	1.0
	Any	Survey of pregnant women unless significant potential for bias (e.g., self-selection or exclusions)	
	≥250	AND	
		Sample likely to be representative of general population or target population	
		-Random sample based on population or census list or registry	
		-Random or consecutive persons attending a clinic or site	
		- Pregnant women tested at antenatal clinics or as part of infant vaccination study	
		"Healthy controls" in controlled study if above criteria are met	
		Potential for bias is limited	
		AND	
		Sample likely to be representative of general population or target population (e.g., pregnant women, villagers, visa applicants)	
		-Stratified, multistage, random sample	
	<250	-OR percent invited who participate is reported and ≥70%	
		Demographics of subjects reported (age and sex)	
		Percent invited who participate is reported and ≥70%	
		Potential for bias is limited	
		AND	
		No description of sampling method (i.e., how subjects were selected for participation)	
		No description of eligibility criteria	
C	Any	Reported as a "convenience sample"	0.8
		(A "random sample" with no description of sampling method = a convenience sample)	
		Sample not likely to be representative of the general population	

Domain	Sample size	Description	Scores
		Potential for bias (e.g., opportunity for self-selection as in a free screening program)	

*Note: adapted from Kowdley et al's systematic review [2].

Table S9: Weighted HBV prevalence of studies among pregnant women in the EU/EEA and UK

Country	*Number of studies*	*Total sample*	*(%) Prevalence*	*(%) 95CI*
Bulgaria	1	2700	2.26	2.25-2.26
Denmark	2	201353	0.26	0.26-0.26
Finland	2	102894	0.13	0.13-0.13
France	1	1112	0.18	0.17-0.18
Germany	2	24066	0.69	0.69-0.69
Greece	6	10246	2.50	2.49-2.50
Ireland	1	24008	0.21	0.20-0.21
Italy	6	72793	0.90	0.90-0.90
Netherlands	15	2696777	0.31	0.31-0.31
Norway	1	1668	0.06	0.05-0.06
Poland	1	460	0.87	0.86-0.87
Romania	1	531	5.08	5.06-5.10
Slovakia	2	24537	2.21	2.21-2.21
Spain	4	34512	0.52	0.52-0.52
UK	4	193480	0.59	0.59-0.59

Table S10: Weighted HBV prevalence of identified studies among first-time blood donors in the EU/EEA and UK

Country	*Number of studies*	*Total sample*	*(%) Prevalence*	*(%)95CI*
Austria	7	318919	0.08	0.08-0.08
Belgium	19	1040377	0.07	0.07-0.07
Bulgaria	5	163162	3.96	3.96-3.96
Croatia	10	132383	0.14	0.14-0.14
Cyprus	4	42712	0.15	0.15-0.15
Czech	11	487040	0.05	0.05-0.05
Denmark	9	242993	0.03	0.03-0.03
Estonia	9	64079	0.14	0.14-0.15
Finland	14	228699	0.07	0.07-0.07
France	24	9998185	0.03	0.03-0.03
Germany	11	5787918	0.11	0.11-0.11

Country	*Number of studies*	*Total sample*	*(%) Prevalence*	*(%)95CI*
Greece	11	707697	1.33	1.33-1.33
Hungary	9	473559	0.10	0.10-0.10
Iceland	5	9265	0.06	0.06-0.06
Ireland	9	143912	0.02	0.02-0.02
Italy	11	5972413	0.18	0.18-0.18
Latvia	3	27644	0.39	0.39-0.39
Lithuania	7	446806	0.18	0.18-0.18
Luxembourg	3	3925	0.17	0.17-0.17
Malta	5	11644	0.24	0.24-0.24
Netherlands	10	335085	0.04	0.04-0.04
Norway	9	145141	0.03	0.03-0.03
Poland	6	1244841	0.39	0.39-0.39
Portugal	5	198498	0.10	0.10-0.10
Romania	6	568842	2.71	2.71-2.71
Slovakia	11	353395	0.09	0.09-0.09
Slovenia	5	56219	0.09	0.09-0.09
Spain	9	2271299	0.15	0.15-0.15
Sweden	8	339673	0.0349	0.03-0.03
UK	10	2214993	0.03	0.03-0.03

Table S11*: *Weighted HBV prevalence of identified studies in the general population in the EU/EEA and UK

Country	*Number of studies*	*Total sample*	*(%) Prevalence*	*(%) 95CI*
Belgium	2	3326	0.69	0.69-0.69
Bulgaria	1	865	3.93	3.91-3.94
Croatia	2	2268	0.92	0.92- 0.93
Czech	1	2644	0.34	0.33-0.34
France	4	38767987	0.80	0.80-0.80
Germany	3	30311	0.47	0.47-0.48
Greece	4	6346	2.44	2.44-2.45
Hungary	1	1066	0.38	0.37-0.38
Ireland	2	4013	0.10	0.10-0.10
Italy	13	42263	2.06	2.05-2.06
Netherlands	4	10464	0.25	0.25-0.25
Poland	3	13601	0.92	0.92-0.92
Portugal	2	2158	1.23	1.22-1.23
Romania	2	14386	4.49	4.49-4.50
Slovakia	4	8503	0.56	0.56-0.56
Spain	8	65529	0.69	0.69-0.69
UK	4	2185129	1.28	1.28-1.28

Table S12: Weighted HBV prevalence of identified studies among migrants in the EU/EEA and UK

Country	Number of studies	Total sample	(%) Prevalence	(%) 95CI
Belgium	3	1967	3.51	3.50-3.51
Denmark	1	92	6.52	6.46-6.57
Finland	1	22016	1.40	1.39-1.40
France	7	7523	5.52	5.52-5.53
Germany	10	103524	3.75	3.75-3.75
Greece	6	6391	5.00	4.99-5.00
Italy	25	25072	10.94	10.93-10.94
Malta	1	500	6.20	6.17-6.22
Netherlands	9	11884	4.06	4.06-4.06
Norway	2	430	0.93	0.92-0.94
Spain	12	12409	12.24	12.24-12.25
Sweden	1	189	1.59	1.57-1.60
UK	14	419160	0.37	0.37-0.37

Table S13: Weighted HBV prevalence of identified studies among men who have sex with men in the EU/EEA

Country	*Number of studies*	*Total sample*	*(%) Prevalence*	*(%)95CI*
Belgium	1	217	2.00	1.98-2.01
Croatia	1	87	3.40	3.36-3.43
Estonia	1	148	3.40	3.37-3.42
France	2	5590	0.40	0.40-0.41
Latvia	1	150	2.00	1.97-2.02
Netherlands	1	376	0.50	0.49-0.50

Table S14: Weighted HBV prevalence of identified studies among people in prison in the EU/EEA and UK

Country	*Number of studies*	*Total sample*	*(%) Prevalence*	*(%)95CI*
Bulgaria	1	258	25.19	25.14-25.24
Croatia	2	3488	1.28	1.28-1.29
Finland	1	383	0.52	0.51-0.53
France	1	347	0.58	0.57-0.59
Hungary	1	4894	1.47	1.47-1.47
Ireland	1	777	0.26	0.25-0.26

Country	*Number of studies*	*Total sample*	*(%) Prevalence*	*(%)95CI*
Italy	1	973	6.68	6.66-6.69
Luxembourg	1	115	6.96	6.91-7.00
Portugal	1	151	0.66	0.65-0.67
Romania	1	197	10.66	10.61-10.70
UK	2	640	1.56	1.55-1.57

Table S15: Results of the risk of bias assessment for the general population

Country	*Authors*	*Publication Year*	*Age bias (0or 1)*	*Gender bias (0 or 1)*	*Sampling method (0, 1 or2)*	*Population coverage (0, 1 or 2)*	*Total Score (0-6)*
Sweden	Christenson et al.	1997	1	1	2	2	6
France	Brouard et al.	2019	1	1	1	2	5
Greece	Touloumi et al.	2017	1	1	2	1	5
Netherlands	Zuure et al.	2019	1	1	2	1	5
Poland	Stępień et al.	unpublished	1	1	1	2	5
Spain	Cuadrado et al.	2020	1	1	0	2	4
UK	Dhillon et al.	2020	0	1	2	1	4
UK	Ireland et al.	2019	1	1	0	2	4
Greece	Karatapanis et al.	2018	1	1	0	1	3
Slovakia	Drazilova et al.	2018	1	0	1	1	3
Spain	Rodriguez-Tajes et al.	2020	1	0	1	1	3
Italy	Caccamo et al.	2019	0	1	0	1	2
Netherlands	Heil et al.	2018	0	1	0	1	2
Slovakia	Macejova et al.	2020	1	0	0	1	2
Spain	Otero et al.	2018	0	1	0	1	2
Iceland	Briem et al.	1990	0	0	0	2	2
Spain	Limia sánchez et	2021	0	0	0	2	2
Sweden	Hoffman et al.	2000	0	1	0	0	1
Denmark	Hansen et al.	2013	1	0	0	0	1

Country	*Authors*	*Publication Year*	*Age bias (0or 1)*	*Gender bias (0 or 1)*	*Sampling method (0, 1 or2)*	*Population coverage (0, 1 or 2)*	*Total Score (0-6)*
Ireland	O'Connor et al.	2018	0	0	0	1	1
Slovakia	Slovak Vaccinolog	2019	0	0	0	1	1

Table S16: Results of the risk assessment for migrants

Country	*Authors*	*Publication Year*	*Risk of bias (0.8,1,1.2)*
Netherlands	Zuure et al.	2019	1.2
Belgium	Ho et al.	2020	1
Belgium	Koc et al.	2020	1
Finland	Tiittala et al.	2018	1
France	Boyd et al.	2018	1
France	Roudot-Thoraval et	2017	1
France	Larsen et al.	2017	1
Germany	Ackermann et al.	2018	1
Greece	Toulomi et al	2017	1
Italy	Coppola et al.	2020	1
Italy	Cuomo et al.	2019	1
Italy	Sagnelli et al.	2018	1
Spain	Salas-Coronas et	2018	1
UK	Flanagan et al.	2019	1
UK	Kelly et al.	2020	1
France	Brouard et al.	2020	0.8
France	Brouard et al.	2020	0.8
Ireland	Brennan et al.	2013	0.8
Italy	Del Pinto et al.	2018	0.8

Country	*Authors*	*Publication Year*	*Risk of bias (0.8,1,1.2)*
Italy	Scotto et al.	2018	0.8
Italy	Scotto et al.	2019	0.8
Italy	Scribano et al.	2019	0.8
Netherlands	Bil et al.	2018	0.8
Spain	Valerio et al.	2008	0.8

Table S17: Results of the risk assessment for pregnant women

Country	*Authors*	*Publication Year*	*Sampling method (0, 1 or 2)*	*Population coverage (0, 1 or 2)*	*Total score (0-3)*
Romania	Popovici et al.	2018	1	2	3
Spain	Ruiz-Extremera et al.	2020	0	2	2
Netherlands	Visser et al.	2019	0	2	2
France	Brouard et al.	2020	0	2	2
Netherlands	Van den Broek et al.	2016	0	2	2
Netherlands	Van den Broek et al.	2016	0	2	2
Netherlands	Van den Broek et al.	2016	0	2	2
Netherlands	Van den Broek et al.	2016	0	2	2
Netherlands	Staritsky et al.	2020	0	2	2
Netherlands	Staritsky et al.	2020	0	2	2
Netherlands	Staritsky et al.	2020	0	2	2
Netherlands	Staritsky et al.	2020	0	2	2
Netherlands	Staritsky et al.	2020	0	2	2
Netherlands	Staritsky et al.	2020	0	2	2
Netherlands	Staritsky et al.	2021	0	2	2

Country	***Authors***	***Publication Year***	***Sampling method (0, 1 or 2)***	***Population coverage (0, 1 or 2)***	***Total score (0-3)***
Ireland	National Perinatal Hepatitis B	2017	0	2	2
Italy	Dalmartello et al	2019	0	1	1
Italy	Giache et al.	2019	0	1	1
Spain	Mur Sierra et al.	2010	0	1	1
Finland	Finnish Institute, Annual statistics	NA	0	0	0
Finland	Finnish Institute, Annual statistics	NA	0	0	0

Table S18: Results of the risk assessment for men who sex with men

Country	***Authors***	***Publication Year***	***Population coverage (0, 1 or 2)***	***Total score (0-2)***
Croatia	Vilibic-cavlek et al.	2018	2	2
Netherlands	Hoornenborg et al.	2018	2	2
France	Vaux et al.	2018	2	2
Latvia	Ķīvīte et al.	2018	2	2
France	Calin et al.	2020	1	1
Estonia	Rüütel et al.	unpublished	1	1
Iceland	Unpublished		0	0
Belgium	Unpublished		0	0

Table S19: Results of the risk assessment for People in prison

Country	***Authors***	***Publication Year***	***Age bias (0or 1)***	***Gender bias (0to 1)***	***PWID bias (0 or 1)***	***Sampling method (0, 1 or 2)***	***Population coverage (0, 1 or 2)***	***Total Score (0-6)***
Sweden	Gahrton et al.	2019	1	1	1	0	2	5
Greece	Sypsa et al.	unpublished data	1	1	1	0	2	5

Latvia	Žabko et al.	2018	1	1	0	0	2	4
Spain	Saiz de la Hoya et al.	2011	0	1	0	1	2	4
Spain	Unpublished	2016	1	1	0	0	2	4
Belgium	Busschots et al.	2020	0	0	1	0	2	3
France	Izquierdo et al.	2019	1	1	1	0	0	3
France	Jacomet et al.	2016	1	1	1	0	0	3
Italy	Sagnelli et al.	2012	0	0	1	0	2	3
Italy	Stasi et al.	2019	1	0	1	0	1	3
Spain	Ferrer-Castro et al.	2012	1	0	1	1	0	3
Poland	unpublished	unpublished	0	0	0	1	0	1

Table S20: Number of identified estimates for the prevalence of chronic hepatitis B (HBsAg) from 2018-2021

Country	First-time blood donors	General Population	Pregnant women	MSM	People in prison	Migrants	Total
Austria	0	0	0	0	0	0	0
Belgium	8	0	0	1	1	2	4
Bulgaria	0	0	0	0	0	0	0
Croatia	1	0	0	0	0	0	0
Cyprus	0	0	0	1	0	0	1
Czech Republic	0	0	0	0	0	0	0
Denmark	0	1	0	0	0	0	1
Estonia	0	0	0	1	0	0	1
Finland	5	0	2	0	0	1	3
France	16	1	1	2	2	5	11
Germany	0	0	0	0	0	1	1
Greece	0	2	0	0	1	1	5
Hungary	0	0	0	0	0	0	0
Iceland	0	1	0	1	0	0	2
Ireland	5	1	1	0	0	1	3
Italy	2	1	2	0	2	7	13
Latvia	0	0	0	1	1	0	2
Liechtenstein	0	0	0	0	0	0	0
Lithuania	1	0	0	0	0	0	0
Luxembourg	0	0	0	0	0	0	0
Malta	0	0	0	0	0	0	0
Netherlands	5	2	12	1	0	2	17
Norway	0	0	0	0	0	0	0
Poland	1	1	0	0	1	0	2
Portugal	0	0	0	0	0	0	0
Romania	0	0	1	0	0	0	1

Country	First-time blood donors	General Population	Pregnant women	MSM	People in prison	Migrants	Total
Slovakia	0	3	0	0	0	0	3
Slovenia	12	0	0	0	0	0	0
Spain	1	4	2	0	3	2	11
Sweden	4	2	0	0	1	0	3
United Kingdom	0	2	0	0	0	2	4

***Table S21**: Sample size of studies presented in figure 2 and 3.*

Estimates included in the figures were sampled from first time blood donors (FTDB), men who have sex with men (MSM), migrants, general population (GP), pregnant women (PW) and prisoner populations.

Group	Country	Year	Sample	HBV prevalence (%)	Author
FTBD	Belgium	Unpublished	53144	0.1	Seroplevance studies
FTBD	Belgium	Unpublished	56220	0.1	Seroplevance studies
FTBD	Belgium	Unpublished	53868	0.1	Seroplevance studies
FTBD	Belgium	Unpublished	54282	0.1	Seroplevance studies
FTBD	Belgium	Unpublished	50575	0.0	Seroplevance studies
FTBD	Belgium	Unpublished	52881	0.1	Seroplevance studies
FTBD	Belgium	Unpublished	59323	0.0	Seroplevance studies
FTBD	Belgium	Unpublished	58933	0.0	Seroplevance studies
FTBD	Finland	NA	13359	0.0	FRC BS, Annual statistics
FTBD	Croatia	2020	NA	0.1	Transfuziološki Vjesnik
FTBD	Finland	NA	14347	0.0	FRC BS, Annual statistics
FTBD	Finland	NA	14739	0.0	FRC BS, Annual statistics
FTBD	Finland	NA	13713	0.0	FRC BS, Annual statistics
FTBD	Finland	NA	15540	0.0	FRC BS, Annual statistics
FTBD	France	2020	862000	0.1	Santé publique France Data (unpublished)
FTBD	France	2005	352348	0.1	Santé publique France Data (unpublished)
FTBD	France	2006	363175	0.1	Santé publique France Data (unpublished)
FTBD	France	2007	395919	0.1	Santé publique France Data (unpublished)
FTBD	France	2008	416367	0.1	Santé publique France Data (unpublished)
FTBD	France	2009	435693	0.1	Santé publique France Data (unpublished)
FTBD	France	2010	368076	0.1	Santé publique France Data (unpublished)
FTBD	France	2011	397010	0.1	Santé publique France Data (unpublished)
FTBD	France	2012	382955	0.1	Santé publique France Data (unpublished)

Group	Country	Year	Sample	HBV prevalence (%)	Author
FTBD	France	2013	357510	0.1	Santé publique France Data (unpublished)
FTBD	France	2014	350333	0.1	Santé publique France Data (unpublished)
FTBD	France	2015	325813	0.1	Santé publique France Data (unpublished)
FTBD	France	2016	279454	0.1	Santé publique France Data (unpublished)
FTBD	France	2017	289427	0.1	Santé publique France Data (unpublished)
FTBD	France	2018	293456	0.0	Santé publique France Data (unpublished)
FTBD	France	2019	289966	0.0	Santé publique France Data (unpublished)
FTBD	Ireland	NA	11508	0.0	IBTS Blood donor screening data
FTBD	Ireland	NA	10237	0.0	IBTS Blood donor screening data
FTBD	Ireland	NA	12762	0.0	IBTS Blood donor screening data
FTBD	Ireland	NA	10704	0.0	IBTS Blood donor screening data
FTBD	Ireland	NA	11480	0.0	IBTS Blood donor screening data
FTBD	Italy	2013	31190	0.3	Romanò et al.
FTBD	Italy	2019	2723639	0.2	Velati et al.
FTBD	Lithuania	2021	292827	0.8	Samanta Grubyte et al.
FTBD	Netherlands	NA	NA	0.0	Staritsky
FTBD	Netherlands	NA	NA	0.0	Staritsky
FTBD	Netherlands	NA	NA	0.0	Staritsky
FTBD	Netherlands	NA	NA	0.0	Staritsky
FTBD	Netherlands	NA	NA	0.0	Staritsky
FTBD	Poland	NA	156447	0.2	Scrofani C, le Tallec D, Rautmann G.
FTBD	Slovenia	2021	6643	0.1	Internal report
FTBD	Slovenia	2020	9324	0.1	Internal report
FTBD	Slovenia	2019	9822	0.0	Internal report
FTBD	Slovenia	2018	9514	0.1	Internal report
FTBD	Slovenia	2017	9548	0.1	Internal report
FTBD	Slovenia	2016	8654	0.0	Internal report
FTBD	Slovenia	2015	9596	0.1	Internal report
FTBD	Slovenia	2014	10369	0.1	Internal report
FTBD	Slovenia	2013	10706	0.1	Internal report
FTBD	Slovenia	2012	12781	0.1	Internal report
FTBD	Slovenia	2011	10608	0.1	Internal report
FTBD	Slovenia	2010	11476	0.1	Internal report
FTBD	Spain	2007	166537	0.2	NA

Group	Country	Year	Sample	HBV prevalence (%)	Author
FTBD	Sweden	NA	38407	0.0	unpublished data, collected data by Public Health Agency of Sweden
FTBD	Sweden	NA	39181	0.0	unpublished data, collected data by Public Health Agency of Sweden
FTBD	Sweden	NA	39946	0.0	unpublished data, collected data by Public Health Agency of Sweden
FTBD	Sweden	NA	NA	0.0	Scrofani C, le Tallec D, Rautmann G
GP	Denmark	2013	1321000	0.2	Hansen et al.
GP	France	2019	6945	0.3	Brouard et al.
GP	Greece	2018	1076	3.8	Karatapanis et al.
GP	Greece	2017	4235	1.7	Touloumi et al.
GP	Italy	2019	316	0.9	Caccamo et al.
GP	Ireland	2018	4588252	0.5	O'Connor
GP	Iceland	1990	366425	0.2	Briem et al.
GP	Netherlands	2019	500	0.4	Zuure et al.
GP	Netherlands	2018	3434	0.3	Heil et al.
GP	Poland	unpublished	5001	0.8	Stępień et al.
GP	Slovakia	2020	403	2.8	Macejova et al.
GP	Slovakia	2019	4128	0.1	Slovak Vaccinological congress
GP	Slovakia	2018	710	2.8	Drazilova et al.
GP	Spain	2018	32203	0.9	Otero et al.
GP	Spain	2020	3328	0.5	Rodriguez-Tajes et al.
GP	Spain	2020	12246	0.6	Cuadrado et al.
GP	Spain	2021	6056	0.1	Limia sánchez et al.
GP	Sweden	2000	5533	0.2	Hoffman et al.
GP	Sweden	1997	3381	0.1	Christenson et al.
GP	United Kingdom	2020	2149933	1.3	Dhillon et al.
GP	United Kingdom	2019	25609	0.1	Ireland et al.
Migrants	Belgium	2020	571	6.8	Ho et al.
Migrants	Belgium	2020	1081	2.4	Koc et al.
Migrants	Finland	2018	22016	1.4	Tiittala et al.
Migrants	France	2018	1090	1.9	Boyd et al.
Migrants	France	2017	2870	7.4	Roudot-Thoraval et al.
Migrants	France	2017	1283	5.1	Larsen et al.
Migrants	France	2020	651	5.7	Brouard et al.
Migrants	France	2020	144	4.9	Brouard et al.
Migrants	Germany	2018	94843	3.3	Ackermann et al.
Migrants	Greece	2017	505	7.3	Toulomi et al.
Migrants	Ireland	2013	10014	4.4	Brennan et al.
Migrants	Italy	2020	3839	9.9	Coppola et al.

Group	Country	Year	Sample	HBV prevalence (%)	Author
Migrants	Italy	2019	304	12.2	Cuomo et al.
Migrants	Italy	2018	1727	9.8	Sagnelli et al.
Migrants	Italy	2018	44	22.7	Del Pinto et al.
Migrants	Italy	2018	195	21.0	Scotto et al.
Migrants	Italy	2019	205	11.2	Scotto et al.
Migrants	Italy	2019	1024	8.2	Scribano et al.
Migrants	Netherlands	2019	2487	2.5	Zuure et al.
Migrants	Netherlands	2018	457	2.2	Bil et al.
Migrants	Spain	2018	523	31.7	Salas-Coronas et al.
Migrants	Spain	2008	791	5.9	Valerio et al.
Migrants	UK	2019	11929	1.1	Flanagan et al.
Migrants	UK	2020	229	0.9	Kelly et al.
MSM	Belgium		217	0.0	Unpublished
MSM	Croatia	2018	87	0.0	Vilibic-cavlek et al.
MSM	Estonia	unpublished	148	0.0	Rüütel et al.
MSM	France	2018	2945	0.0	Vaux et al.
MSM	France	2020	2645	0.0	Calin et al.
MSM	Iceland		300	0.0	Unpublished
MSM	Latvia	2018	150	0.0	Ķīvīte et al.
MSM	Netherlands	2018	376	0.0	Hoornenborg et al.
PW	Finland	NA	51447	0.2	Grey literature
PW	Finland	NA	51447	0.1	Grey literature
PW	France	2020	12775	0.8	Brouard et al.
PW	Ireland	2017	8632	0.3	National Perinatal Hepatitis B Prevention Group
PW	Italy	2019	682	5.1	Giache
PW	Italy	2019	38712	0.9	Dalmartello et al.
PW	Netherlands	2016	185528	0.4	Van den Broek et al.
PW	Netherlands	2019	190140	0.3	Visser et al.
PW	Netherlands	2016	182297	0.3	Van den Broek et al.
PW	Netherlands	2016	174646	0.3	Van den Broek et al.
PW	Netherlands	2016	187586	0.3	Van den Broek et al.
PW	Netherlands	2020	176086	0.3	Staritsky et al.
PW	Netherlands	2020	175927	0.3	Staritsky et al.
PW	Netherlands	2020	176238	0.3	Staritsky et al.
PW	Netherlands	2020	172799	0.3	Staritsky et al.
PW	Netherlands	2020	170461	0.3	Staritsky et al.
PW	Netherlands	2020	171242	0.3	Staritsky et al.
PW	Netherlands	2021	171609	0.3	Staritsky et al.
PW	Romania	2018	531	5.1	Popovici et al.
PW	Spain	2020	21870	0.4	Ruiz-Extremera et al.
PW	Spain	2010	1743	0.4	Mur Sierra et al.
Prisoners	Greece	Unpublished	327	8.3	Sypsa et al
Prisoners	Latvia	2018	1017	6.0	Žabko et al.

Group	Country	Year	Sample	HBV prevalence (%)	Author
Prisoners	Italy	2012	2265	4.4	Sagnelli et al.
Prisoners	Poland	Unpublished	5406	3.5	Unpublished
Prisoners	Spain	2011	342	2.6	Saiz de la Hoya et al.
Prisoners	Spain	2016	5024	2.1	Unpublished
Prisoners	Spain	2012	425	2.1	Ferrer-Castro et al.
Prisoners	Sweden	2019	465	1.9	Gahrton et al.
Prisoners	France	2019	1093	1.9	Izquierdo et al.
Prisoners	Italy	2019	1075	1.9	Stasi et al.
Prisoners	Belgium	2020	456	1.1	Busschots et al.
Prisoners	France	2016	326	0.6	Jacomet et al.

References

1. European Centre for Disease Prevention and Control, *Systematic review on hepatitis B and C prevalence in the EU/EEA*. 2016.
2. Kowdley, K.V., et al., *Prevalence of chronic hepatitis B among foreign-born persons living in the United States by country of origin.* Hepatology, 2012. **56**(2): p. 422-33.
